# Effects of Local Vibration and Foam Rolling on Hip Pain and Function in Mild to Moderate Hip Osteoarthritis: A Randomized Controlled Trial

**DOI:** 10.7759/cureus.83674

**Published:** 2025-05-07

**Authors:** Hisashi Ikutomo, Masatoshi Nakamura, Kenichi Okamura, Keiichi Togomori, Koutatsu Nagai, Norikazu Nakagawa, Kensaku Masuhara

**Affiliations:** 1 Department of Rehabilitation, Masuhara Clinic, Osaka City, JPN; 2 Faculty of Rehabilitation Sciences, Department of Physical Therapy, Nishikyushu University, Saga, JPN; 3 Department of Physical Therapy, School of Rehabilitation, Hyogo Medical University, Kobe, JPN; 4 Department of Orthopaedics, Masuhara Clinic, Osaka City, JPN

**Keywords:** foam roller, hip osteoarthritis, hip pain, physical therapy, rehabilitation, vibration

## Abstract

Background: Local vibration and foam rolling effectively reduce muscle soreness and improve the function of damaged muscles. However, their efficacy in decreasing hip pain and improving function in patients with hip osteoarthritis remains unclear. This study aimed to investigate the effects of local vibration and foam rolling on hip pain and function in patients with mild to moderate hip osteoarthritis.

Methods: Thirty-five patients with mild to moderate hip osteoarthritis were randomly assigned to the local vibration, foam rolling, and exercise groups. All patients underwent the assigned interventions at home daily for four weeks. The primary outcome was hip pain intensity assessed by a visual analog scale. The secondary outcomes were physical functions, including range of motion of the hip joint, physical function, such as gait velocity and timed up-and-go test, physical activity, pain catastrophizing scale, Harris hip score, and hip disability and osteoarthritis outcome score.

Results: After four weeks of intervention, significant group × time interactions were observed for hip pain during walking (p = 0.02, partial η^2^ = 0.16) and hip adduction range of motion (p = 0.02, partial η^2^ = 0.26). Post-hoc analysis showed greater improvements in hip pain and range of motion in the local vibration and foam rolling groups compared to the exercise group. No significant interactions were found for the other outcomes.

Conclusion: These results suggested that local vibration and foam rolling effectively reduced hip pain and improved hip adduction range of motion in patients with mild to moderate hip osteoarthritis. However, there was no additional effect on other physical functions.

## Introduction

Hip osteoarthritis (OA) is one of the most common degenerative joint diseases, primarily affecting articular cartilage and surrounding tissues [1,2]. Patients with hip OA frequently experience musculoskeletal pain, functional disability, and reduced quality of life [3-5]. A systematic review and meta-analysis found that the prevalence of radiographic hip OA was approximately 8.6% worldwide, with the prevalence increasing with age [6]. The estimated lifetime risk of symptomatic hip OA is one in four people by the age of 85 years [7]. The incidence of hip OA is increasing with the aging of the population, making it a major public health concern worldwide.

In the nonsurgical management of hip OA, clinical practice guidelines commonly recommend muscle strengthening, exercise (EX), education, and weight loss [3,8,9]. In addition, the guidelines conditionally recommend that manual therapy could improve joint mobility impairment or pain for patients with mild to moderate hip OA [3,9]. However, the details of effective exercises for reducing hip pain and functional disability in patients with hip OA remain inconclusive. Therefore, effective interventions are required to treat and prevent hip pain and disability in patients with hip OA.

Foam rolling (FR) is a self-applied massage technique that uses a foam roller. During FR, individuals employ their body weight to exert pressure on soft tissue through a rolling motion over a foam roller. FR reduces muscle soreness in healthy individuals after exercise-induced muscle damage [10-12]. Furthermore, a previous retrospective study suggested that FR may effectively reduce hip pain in patients with hip OA [13]. In contrast, previous studies have reported that local vibration (LV) delivered via vibration rollers-foam rollers equipped with an integrated vibration function-effectively reduces muscle soreness and improves functional disability after exercise-induced muscle damage [14,15]. Additionally, static compression using a foam roller, wherein individuals apply body weight to exert pressure on the muscle with or without performing rolling movement, could improve muscle soreness and function [14]. This intervention is simpler than FR and more applicable to various individuals, including older people and those with impaired upper extremity function. However, to the best of our knowledge, no previous studies have investigated the effects of LV using vibration rollers on hip pain and function in patients with hip OA.

Therefore, we aimed to compare the effects of LV using a vibration roller, FR, and lower limb muscle strengthening on hip pain and function in patients with mild to moderate hip OA.

## Materials and methods

Study design

This study followed a participant-blinded randomized controlled trial design with a three-arm parallel-group design. The trial was registered in the University Hospital Medical Information Network Clinical Trials Registry (UMIN000044360), conducted in accordance with the principles of the Declaration of Helsinki, and received approval from the ethics review board of the author’s affiliated institution on June 1, 2021 (#18613-210601).

Participants

We enrolled 35 patients with mild to moderate hip OA who visited an orthopedic clinic in Japan between June 9, 2021 and April 10, 2023. The inclusion criteria were as follows: 1) first-time visit to the clinic; 2) diagnosis of hip OA classified as grade 1 to 3 on the Kellgren and Lawrence radiographic index [16]; 3) pain around the hip joint for more than one month; and 4) average pain intensity of ≥40 on a 100 mm visual analog scale (VAS) while walking during the past week. The exclusion criteria were as follows: 1) age <20 or ≥80 years; 2) hip OA diagnosis of grade 0 or 4 on the Kellgren and Lawrence radiographic index; 3) diagnosis of rheumatoid arthritis, femoral head necrosis, or osteoarthritis other than that of the hip joint; 4) disorders of the nervous system and muscles; 5) history of hip surgery; and 6) history of physical therapy or self-exercise within the last month. All patients were informed of the purpose and procedure of the study and provided written informed consent prior to participation.

We calculated the sample size required for a split-plot analysis of variance (effect size = 0.25, α error = 0.05, and power = 0.80) using G*Power 3.1 software (Heinrich Heine University, Dusseldorf, Germany) based on a previous study [13]. The sample size calculation showed at least eight participants per group.

Randomization and masking

Participants were randomly allocated to one of three intervention groups: LV, FR, or EX. Stratified block randomization technique (four participants per block) was employed, with permuted block randomization executed in each block using a random number table generated in Microsoft Excel (Microsoft Corporation, Redmond, WA, USA). The participants underwent a blinding procedure in which they remained unaware of their group assignment. In addition, to minimize bias, participants were not informed of the study hypothesis, ensuring that they lacked knowledge regarding the expected intervention effects. However, due to the nature of the interventions, blinding the outcome assessors and physical therapists administering the interventions was not feasible.

Baseline characteristics

Baseline characteristics, including sex, age, height, body mass, number of prescribed medications, presence of comorbidities, and symptom duration, were collected using a self-administered questionnaire and structured interviews. Additionally, the Kellgren and Lawrence radiographic index and the Harris hip score were assessed before the intervention [16,17]. The Harris hip score, a disease-specific total hip rating system, utilized a 100-point scale to evaluate pain and functional ability and patients’ ability to walk, climb stairs, and perform daily activities.

Intervention

**Figure 1:**
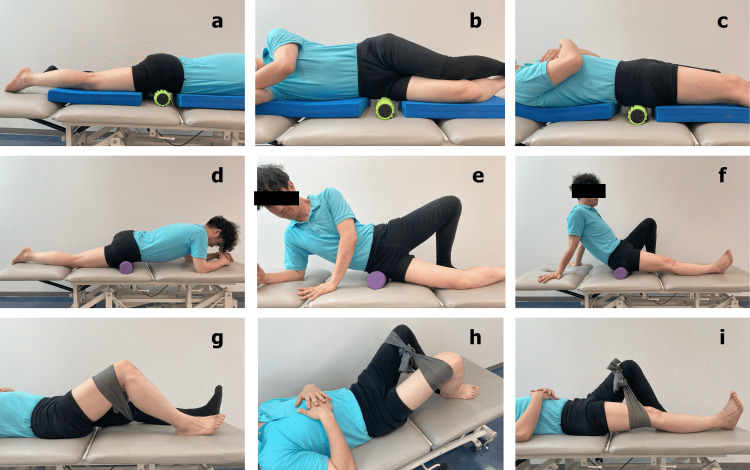
Methods of interventions (a) local vibration on the anterior hip joint, (b) local vibration on the lateral hip joint, (c) local vibration on the posterior hip joint, (d) foam rolling on the anterior hip joint, (e) foam rolling on the lateral hip joint, (f) foam rolling on the posterior hip joint, (g) hip flexion muscle strengthening exercise, (h) hip abduction muscle strengthening exercise, and (i) hip extension muscle strengthening exercise.

The LV group was instructed to apply the LV using a vibration roller (3D Massage Roll MR-001, Dream Factory, Osaka, Japan; dimensions: 85 × 310 mm; weight: 675 g). LV was applied as static compression to soft tissue without rolling movement on the anterior, lateral, and posterior hip joints of the affected side at 45 Hz for two minutes in each position (Figure 1A, 1B, 1C) [14,15].

The FR group was instructed to perform self-massage using a foam roller (Mini Flex Roller; HIROUN, Osaka, Japan; dimensions: 100 mm × 300 mm; weight: 70 g). FR was performed by applying pressure to the soft tissue on the anterior, lateral, and posterior hip joints of the affected side with a rolling motion on a foam roller for two minutes in each position (Figure 1D, 1E, 1F) [12,13].

The EX group was instructed to perform muscle strengthening exercises using a rubber band (Thera-Band Black, Hygenic Corporation, Akron, OH, USA; dimensions: 125 mm × 1750 mm; weight: 76 g). Exercises involved flexion, abduction, and extension of the hip joint on the affected side with 30 repetitions each (Figure 1G, 1H, 1I).

Three physical therapists with 10 or more years of clinical experience were primarily responsible for the rehabilitation of the participants. The participants were instructed to perform the assigned interventions as home exercises daily for four weeks and to record their daily performance. All participants were informed that the intervention would be stopped immediately if any adverse events occurred, such as dizziness, poor mood, increased pain due to muscle damage or neuropathy. The participants were not instructed on home exercise programs other than the assigned interventions, did not attend outpatient physical therapy sessions, and were not prescribed any analgesics during the intervention period.

Outcome assessment

The primary outcome was hip pain intensity using a 100 mm horizontal VAS, which allowed participants to estimate hip pain by marking an X on a 100 mm line (0 mm for no pain and 100 mm for the worst possible pain) [18]. Hip pain intensity was evaluated at rest, during exercise in the supine position, and while walking. Exercise-induced hip pain was evaluated using a straight leg raise, in which the participant elevated the affected limb with the knee extended in the supine position. Participants recorded hip pain before and weekly after the intervention for up to four weeks. The VAS had high test-retest reliability (intraclass correlation coefficient = 0.94) and was correlated with other pain intensity tests [19]. The minimal clinically important improvement was set at 32% for the hip pain VAS score of patients with hip OA [20].

The secondary outcomes were functional outcomes, including passive range of motion (ROM) of the hip joint (flexion, extension, abduction, and adduction), gait velocity, timed up-and-go test result, physical activity, pain catastrophizing scale score, Harris hip score, and health-related quality of life before and at four weeks after the intervention. The passive hip joint ROM was measured using a goniometer while the pelvis was stabilized to prevent rotation or tilting [21]. The gait velocity was measured using a 10-meter standard walking time. Participants were asked to walk 14 meters at a comfortable pace, with the first and last 2 meters excluded from measurements to eliminate periods of acceleration and deceleration. The timed up-and-go test measures the time taken to stand up from a standard chair, walk 3 meters as quickly and safely as possible, turn, return to the chair, and sit down again [22]. Physical activity was measured using the Japanese version of the International Physical Activity Questionnaire [23], with the total duration (min per week) of walking recorded. Pain catastrophizing was measured using the Japanese version of the pain catastrophizing scale [24,25], which comprises 13 items related to rumination, helplessness, and magnification, each rated on a Likert scale ranging from 0 = “Not at all” to 4 = “All the time”. Health-related quality of life was measured using the Japanese version of the hip disability and OA outcome score [26,27], with each item on the questionnaire (pain: 10; symptoms: 5; activities of daily living (ADL): 17; sport/recreation: 4; quality of life: 4) rated on a Likert scale from 0 to 4. The scores for each domain were normalized from worst to best on a scale of 0-100.

Data analysis

Data are presented as the mean (standard deviation). The effects of interventions on outcome measurements within the groups were compared using a general linear model (GLM) with a split-plot design. The effects of the interventions on the outcome measurements in the groups were compared using a GLM with a split-plot design. If the GLM for hip pain intensity showed a significant group × time interaction, simple main effects were interpreted separately using repeated-measures analysis of variance (time) and one-way analysis of variance (group). Multiple comparisons with the Bonferroni correction post-hoc test (group) and unpaired t-test (time) were conducted whenever the main effects of time and group revealed significance. For functional outcome measurements other than hip pain intensity, if the GLM showed a significant group × time interaction, simple main effects were interpreted separately using the paired t-test (time) and unpaired t-test (group).

The differences were considered statistically significant at p < 0.05. All statistical analyses were conducted using SPSS version 28.0 (IBM Japan Inc., Tokyo, Japan).

## Results

**Figure 2:**
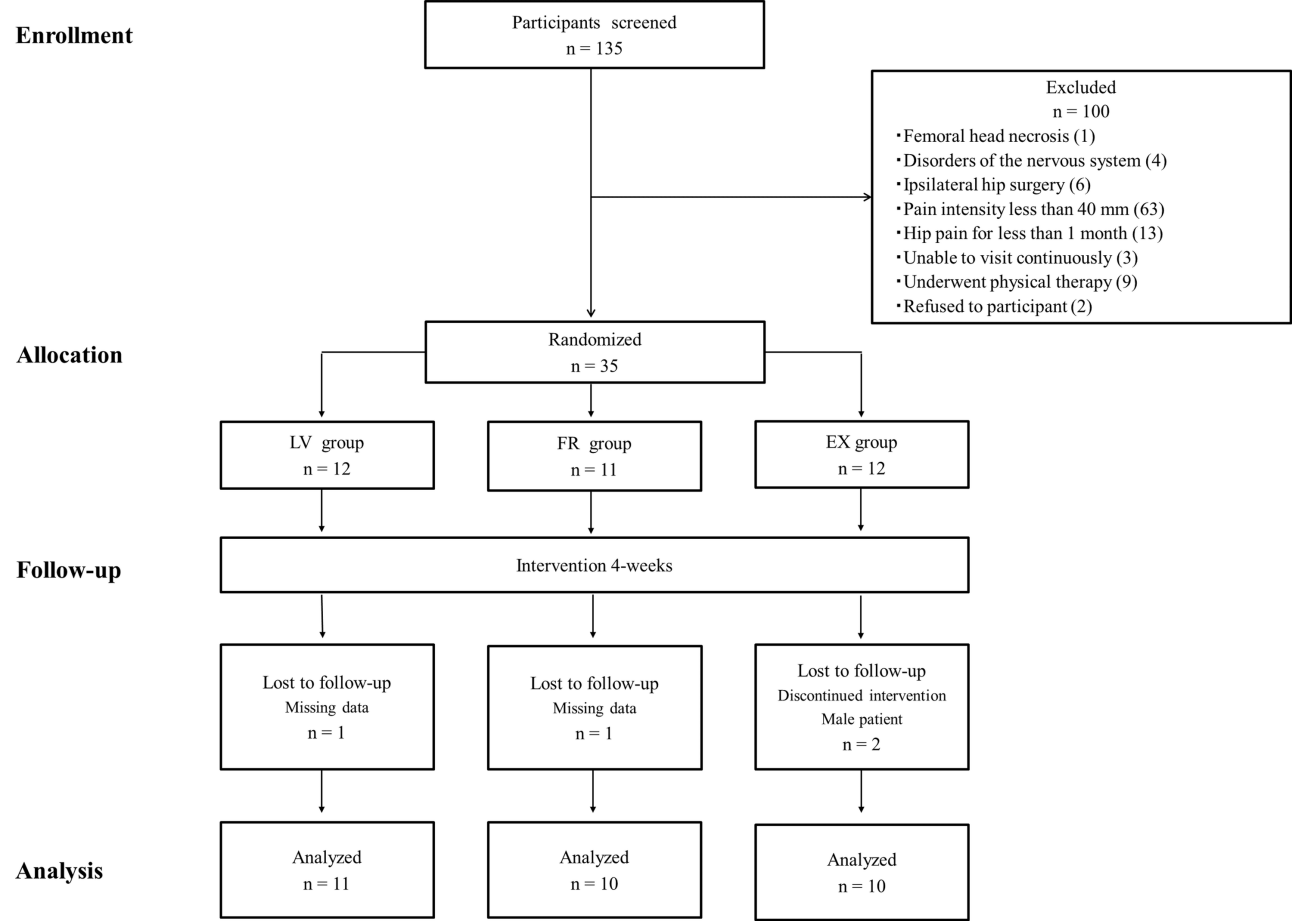
Consolidated Standards of Reporting Trials flow diagram FR: Foam rolling, EX: Exercise, LV: Local vibration

Among the 35 patients with mild to moderate hip OA, four patients with missing follow-up data or an insufficient number of intervention days (less than half of the intervention period) and male patients (n = 1) were excluded (Figure 2). Given the small number of male patients (n = 1), we analyzed only female patients to avoid possible confounding factors stemming from an uneven sex distribution. Hence, 31 (89%) female patients with an average age of 53.1 (12.5) years who completed all measurements were included in the analysis. No dropouts or adverse events occurred during the four-week follow-up in either group. Interventions were performed for a mean (standard deviation) of 22.1 (4.0), 24.1 (3.2), and 21.5 (3.0) days in the LV, FR, and EX groups, respectively (p = 0.260). The baseline characteristics and outcome measurements of the three groups are presented in Table 1 and Table 2.

**Table 1:** Baseline characteristics of the three groups LV: Local vibration, FR: Foam rolling, EX: Exercise, K&L: Kellgren & Lawrence, HHS: Harris hip score, IQR: Interquartile range. Results are expressed as mean (standard deviation) unless otherwise indicated.

Parameter	LV group (n = 11)	FR group (n = 10)	EX group (n = 10)
Age, years	49.8 (13.1)	54.4 (11.6)	55.3 (12.6)
Height, cm	156.5 (5.6)	158.0 (4.4)	159.1 (3.4)
Body mass, kg	51.6 (6.6)	54.4 (7.0)	55.1 (6.3)
Body mass index, kg/m^2^	21.2 (3.2)	21.8 (2.5)	21.7 (2.0)
Analgesic medicines, n (%)	2 (18)	0 (0)	1 (10)
Comorbidities, n (%)			
None	8 (73)	6 (60)	6 (60)
1 or 2	3 (27)	4 (40)	3 (30)
≥ 3	0 (0)	0 (0)	1 (10)
K&L grade, n			
Involved limb (1/2/3)	5/4/2	4/2/4	3/3/4
Uninvolved limb (0/1/2/3)	3/7/1/0	1/7/2/0	2/3/4/1
Duration of symptoms, n (%)			
≥ 1 month but < 3 months	0 (0)	0 (0)	1 (10)
≥ 3 months but < 1 year	8 (73)	5 (50)	5 (50)
≥ 1 year but < 5 year	3 (27)	5 (50)	4 (40)
≥ 5 years	0 (0)	0 (0)	0 (0)
Total HHS, point (IQR)	77 (10.5)	73 (16.5)	72 (21.3)

**Table 2:** Baseline functional outcomes in the three groups LV: Local vibration, FR: Foam rolling, EX: Exercise, VAS: Visual analog scale, ROM: Range of motion, TUG: Timed up and go, IPAQ: International Physical Activity Questionnaire, PCS: Pain catastrophizing score, IQR: Interquartile range, HOOS: Hip disability and osteoarthritis outcome score. Results are expressed as mean (standard deviation) unless otherwise indicated.

Parameter	LV group (n = 11)	FR group (n = 10)	EX group (n = 10)
Hip pain VAS, mm			
Rest	22.6 (22.5)	24.0 (26.0)	16.4 (18.9)
Exercising	27.0 (24.5)	20.3 (20.0)	26.8 (23.1)
Walking	63.5 (7.3)	63.4 (15.7)	56.7 (12.0)
ROM of the hip, degree			
Flexion	106.8 (14.8)	108.5 (11.4)	100.5 (8.2)
Extension	14.1 (5.1)	15.0 (6.3)	11.5 (3.9)
Abduction	34.5 (6.2)	35.5 (4.7)	34.0 (8.0)
Adduction	10.0 (6.4)	8.5 (4.5)	10.0 (5.5)
Gait velocity, m/s	1.32 (0.25)	1.34 (0.23)	1.25 (0.15)
TUG, s	6.8 (1.4)	6.3 (0.9)	6.9 (1.3)
IPAQ gait, min/week	379 (361)	603 (730)	280 (347)
PCS, point (IQR)	31.0 (7.5)	31.5 (24.5)	29.0 (9.0)
Total HOOS, % (IQR)	56.3 (15.0)	51.6 (20.9)	52.8 (11.4)

Hip pain intensity on walking, the primary outcome measure of this study, showed a significant group × time interaction (p = 0.02, partial η^2^ = 0.16) (Table 3). Following the interventions, hip pain intensity during walking was significantly improved in the LV and FR groups compared to the EX group. In the LV group, the mean (standard deviation) hip pain VAS scores at baseline and at four weeks were 63.5 (7.3) and 16.4 (18.8) mm, respectively. In the FR group, the hip pain VAS scores at baseline and at four weeks were 63.4 (15.7) and 17.1 (17.4) mm, respectively. In contrast, the EX group showed hip pain VAS on walking at baseline and at four weeks of 56.7 (12.0) and 40.4 (28.7) mm, respectively. Although the main effects of time in the LV and FR groups showed significant changes (both p < 0.001), the main effect of time in the EX group did not change significantly (p = 0.21). A ≥32% improvement in the hip pain VAS score on walking, which is considered the minimal clinically important improvement, was noted in nine (82%) patients in the LV group; nine (90%) in the FR group; and five (50%) in the EX group. There were no significant group × time interactions for hip pain intensity on rest and exercise (p = 0.54 and 0.60, partial η2 = 0.05, and 0.05, respectively).

**Table 3:** Hip pain VAS scores from pre-intervention to four weeks post-intervention in the three groups VAS: Visual analog scale, LV: Local vibration, FR: Foam rolling, EX: Exercise, ANOVA: Analysis of variance, PRE: Pre-intervention, POST: Post-intervention. Results are expressed as mean (standard deviation), unless otherwise indicated. The split-plot ANOVA results (T: time effect, G X T: group × time interaction effect; F-value), as part of the general linear model, and partial η2 (ηp2) are shown in the right column. Statistical significance was set at p < 0.05.

	LV group (n = 11)					FR group (n = 10)					EX group (n = 10)					ANOVA results ^b^		
Hip pain VAS (mm)	PRE	POST				PRE	POST				PRE	POST				P value, F value, η_p_^2^		
		1 week	2 weeks	3 weeks	4 weeks		1 week	2 weeks	3 weeks	4 weeks		1 week	2 weeks	3 weeks	4 weeks			
Resting	22.6 (22.5)	24.0 (24.1)	27.8 (27.0)	23.4 (26.6)	13.2 (20.2)	24.0 (26.0)	19.2 (23.6)	18.2 (21.3)	11.2 (16.8)	8.0 (18.8)	16.4 (18.9)	21.5 (27.9)	20.1 (29.6)	21.3 (28.2)	16.6 (22.7)	T: p = 0.124	F = 2.23	η_p_^2 ^= 0.074
																G X T: p = 0.544	F = 0.756	η_p_^2^ = 0.051
Exercising	27.0 (24.5)	39.1 (28.3)	32.2 (29.1)	27.5 (27.4)	16.7 (22.6)	20.3 (20.0)	18.4 (22.1)	16.1 (20.8)	13.5 (19.0)	8.3 (15.3)	26.8 (23.1)	30.9 (27.7)	26.4 (27.6)	25.6 (28.1)	23.2 (27.2)	T: p = 0.022,	F = 4.02	η_p_^2 ^= 0.126
																G X T: p = 0.597	F = 0.706	η_p_^2^ = 0.048
Walking	63.5 (7.3)	50.9 (15.5)	41.9 (20.7)	31.5 (20.7)	16.4 (18.8)	63.4 (15.7)	34.6 (21.9)	29.1 (16.5)	20.5 (17.4)	17.1 (17.4)	56.7 (12.0)	49.5 (23.2)	40.2 (31.5)	42.5 (30.0)	40.4 (28.7)	T: p < 0.001	F = 26.8	η_p_^2 ^= 0.489
																G X T: p = 0.024	F = 2.7	η_p_^2^ = 0.162

A significant group × time interaction was observed for hip adduction ROM (p = 0.02, partial η^2^ = 0.26) (Table 4). After the interventions, hip adduction ROM was significantly improved in the LV and FR groups compared to the EX group. Although the main effects of time in the LV and FR groups showed significant changes (both with p = 0.01), the main effect of time in the EX group did not change significantly over time (p = 0.59). Notably, no significant group × time interactions were observed for hip flexion, extension, and abduction ROM; gait velocity; timed up-and-go test result; physical activity; pain catastrophizing scale score; Harris hip score; and hip disability and OA outcome score.

**Table 4:** Functional outcomes from pre-intervention to four weeks post-intervention in the three groups LV: Local vibration, FR: Foam rolling, EX: Exercise, ANOVA: Analysis of variance, PRE: Pre-intervention, POST: Post-intervention, ROM: Range of motion, TUG: Timed up and go, IPAQ: International Physical Activity Questionnaire, IQR: Interquartile range, PCS: Pain catastrophizing score, HHS: Harris hip score. Results are expressed as the mean (standard deviation) unless otherwise indicated. The split-plot ANOVA results (T: time effect, G X T: group × time interaction effect; F-value), as part of the general linear model, and partial η2 (ηp2) are shown in the right column. Statistical significance was set at p < 0.05.

Parameter	LV group (n = 11)		FR group (n = 10)		EX group (n = 10)		ANOVA results ^b^		
	PRE	POST	PRE	POST	PRE	POST	p value, F value, η_p_^2^		
ROM, degree									
Flexion	106.8 (14.8)	111.4 (9.3)	108.5 (11.4)	112.0 (10.0)	100.5 (8.2)	100.0 (9.7)	T: p = 0.04	F = 4.78	η_p_^2 ^= 0.15
							G _X_ T: p = 0.19	F = 1.78	η_p_^2^ = 0.11
Extension	14.1 (5.1)	14.5 (5.0)	15.0 (6.3)	16.0 (6.2)	11.5 (3.9)	11.5 (4.5)	T: p = 0.19	F = 1.81	η_p_^2 ^= 0.06
							G _X_ T: p = 0.54	F = 0.62	η_p_^2^ = 0.04
Abduction	34.5 (6.2)	36.8 (4.4)	35.5 (4.7)	37.0 (4.6)	34.0 (8.0)	34.0 (8.0)	T: p = 0.10	F = 2.91	η_p_^2 ^= 0.09
							G _X_ T: p = 0.45	F = 0.82	η_p_^2^ = 0.06
Adduction	10.0 (6.4)	13.2 (5.3)	8.5 (4.5)	12.0 (5.6)	10.0 (5.5)	9.5 (5.2)	T: p = 0.001	F = 12.74	η_p_^2 ^= 0.31
							G _X_ T: p = 0.02	F = 4.85	η_p_^2^ = 0.26
Gait velocity, m/s	1.32 (0.25)	1.40 (0.21)	1.34 (0.23)	1.43 (0.21)	1.25 (0.15)	1.36 (0.17)	T: p < 0.001	F = 15.03	η_p_^2 ^= 0.35
							G _X_ T: p = 0.96	F = 0.05	η_p_^2^ = 0.003
TUG, s	6.8 (1.4)	6.4 (1.4)	6.8 (0.9)	6.4 (0.8)	6.9 (1.3)	6.4 (0.8)	T: p = 0.01	F = 7.64	η_p_^2 ^= 0.21
							G _X _T: p = 0.61	F = 0.50	η_p_^2^ = 0.04
IPAQ gait, min/week (IQR)	300 (360)	300 (240)	330 (548)	390 (495)	190 (183)	255 (263)	T: p = 0.47	F = 0.55	η_p_^2 ^= 0.02
							G _X _T: p = 0.54	F = 0.64	η_p_^2^ = 0.04
PCS, point (IQR)	31.0 (7.5)	20.0 (15.0)	31.5 (24.5)	21.5 (18.0)	29.0 (9.0)	23.5 (11.8)	T: p < 0.001	F = 15.53	η_p_^2 ^= 0.36
							G _X _T: p = 0.24	F = 1.51	η_p_^2^ = 0.10
HHS, point (IQR)	77.0 (10.5)	88.0 (15.0)	73.0 (16.5)	93.5 (14.0)	72.0 (21.3)	85.5 (31.5)	T: p < 0.001	F = 23.35	η_p_^2 ^= 0.46
							G _X _T: p = 0.13	F = 2.16	η_p_^2^ = 0.13

## Discussion

To the best of our knowledge, this study is the first randomized controlled trial on the effects of LV and FR on hip pain and function in patients with mild to moderate hip OA. Compared to muscle strengthening using a rubber band, LV using a vibration roller and FR using a foam roller improved hip pain during walking and hip adduction ROM. This is the first prospective study to demonstrate the effectiveness of LV and FR as self-interventions for reducing hip pain and improving hip adduction ROM in patients with mild to moderate hip OA.

Both LV and FR were effective in reducing muscle soreness after exercise-induced muscle damage in healthy adults [11,12,14,15]. Similarly, they may have reduced muscle pain around the hip joint in this study. Patients with hip OA often experience pain not only in the hip joint but also at multiple other locations around it. Moreover, 40-70% of patients with hip OA have buttocks and great trochanter pain [28,29]. This pain may not be attributed to intra-articular pathologies but rather to muscle soreness and perceived fatigue in the gluteus muscle group, chronic inflammation via great trochanteric pain syndrome, and abnormal neovessels [30,31]. Therefore, many patients with hip OA may experience hip pain, affecting the hip joint and the surrounding area, and LV and FR may be effective in improving extra-articular pathologies.

Although the exact mechanism of hip pain reduction during LV and FR performance is unclear, previous studies have suggested that the gate control theory of pain and alterations in the parasympathetic nervous system may be related to improvements in pain sensitivity [32,33]. In addition, both LV and FR interventions were effective in increasing the pain pressure threshold [14,33,34] and reducing inflammation [35,36]. Moreover, LV healing improves after muscle injury [37,38]. These mechanisms may explain the reduced hip pain observed in patients with hip OA following LV and FR.

In this study, the LV and FR muscles were more effective in improving hip adduction range in patients with hip OA than muscle strengthening training. Both LV and FR were effective in improving ROM in healthy adults [39,40]. Furthermore, both LV and FR were performed on the soft tissues in the anterior, lateral, and posterior hip joints, which may have a greater effect on the gluteus muscle group. Thus, the LV and FR may have improved hip adduction ROM because of the improved flexibility of the gluteus muscle group.

Muscle strengthening and exercises are recommended for patients with hip OA [3,8,9]. However, the effects of muscle strengthening and exercise on hip pain in patients with hip OA have not yet been established. In this study, LV and FR exercise reduced hip pain during walking compared to muscle strengthening. Both LV and FR are easy to perform and can be continued as self-care methods. Therefore, LV and FR have the potential to emerge as conservative therapeutic options for hip pain in patients with mild to moderate hip OA. Personal preference, accessibility, and affordability are likely to influence the choice between LV and FR in individual patients.

This study has some limitations. First, this study was single-blind; the outcome assessors and physical therapists who prescribed the intervention program were not blinded to group allocation. Second, the small sample size limits the generalizability of the results of this study. Male patients were excluded due to their small sample size. Additionally, this study included patients with mild to moderate hip OA and excluded those with end-stage hip OA. Therefore, further investigations with larger sample sizes are necessary to clarify the effectiveness of LV and FR therapy on hip pain and function in those with all-stage hip OA. Third, it was not possible to include a control group without any type of therapy. All participants had to visit our clinic for hip joint OA treatment, and they were not allowed to miss any interventions. Fourth, all interventions in this study were carried out with home exercises without standardized supervision. This limits the reproducibility and generalizability of the results. Finally, the LV and FR muscles improved hip pain during walking and hip adduction ROM after four weeks of intervention; however, the sustained effect post-intervention remains unclear. Future studies with longer follow-up periods are needed to examine the effects of LV and FR in patients with hip OA.

## Conclusions

We investigated the effects of LV, FR, and EX on hip pain and function in patients with mild to moderate hip OA in a randomized controlled clinical trial. Compared to EX, LV and FR improved hip pain during walking and hip adduction ROM. Therefore, LV and FR could become new self-care methods for patients with mild to moderate hip OA.
